# Lysophospholipid Growth Factors and Their G Protein-Coupled Receptors in Immunity, Coronary Artery Disease, and Cancer

**DOI:** 10.1100/tsw.2002.124

**Published:** 2002-02-06

**Authors:** Edward J. Goetzl, Markus Graeler, Mei-Chuan Huang, Geetha Shankar

**Affiliations:** Department of Medicine, University of California, UB8B, 533 Parnassus at 4th, San Francisco, CA 94143-0711, USA; Ceretek, LLC, Suite 200, 1501 Harbor Bay Parkway, Alameda, CA 94502, USA

**Keywords:** lysophosphatidic acid, sphingosine 1-phosphate, serum response element, T cells, chemotaxis, cytokines, hypoxia, apoptosis, ovarian cancer, cardioprotection

## Abstract

The physiological lysophospholipids (LPLs), exemplified by lysophosphatidic acid (LPA) and sphingosine 1-phosphate (S1P), are omnific mediators of normal cellular proliferation, survival, and functions. Although both LPA and S1P attain micromolar concentrations in many biological fluids, numerous aspects of their biosynthesis, transport, and metabolic degradation are unknown. Eight members of a new subfamily of G protein-coupled LPA/S1P receptors, originally termed Edg Rs, bind either LPA or S1P with high affinity and transduce a series of growth-related and/or cytoskeleton-based functional responses. The most critical areas of LPL biology and pathobiology are neural development and neurodegeneration, immunity, atherosclerosis and myocardial injury, and cancer. Data from analyses of T cells established two basic points: (1) the plasticity and adaptability of expression of LPA/S1P Rs by some cells as a function of activation, and (2) the role of opposing signals from two different receptors for the same ligand as a mechanism for fine control of effects of LPLs. In the heart, LPLs may promote coronary atherosclerosis, but are effectively cytoprotective for hypoxic cardiac myocytes and those exposed to oxygen free radicals. The findings of production of LPA by some types of tumor cells, overexpression of selected sets of LPA receptors by the same tumor cells, and augmentation of the effects of protein growth factors by LPA have suggested pathogenetic roles for the LPLs in cancer. The breadth of physiologic and pathologic activities of LPLs emphasizes the importance of developing bioavailable nonlipid agonists and antagonists of the LPA/S1P receptors for diverse therapeutic applications.

## INTRODUCTION

Lysophosphatidic acid (LPA), sphingosine 1-phosphate (S1P), and other lysophospholipids (LPLs) are amphipathic products of stimulated cells, which are present in many mammalian physiological fluids at up to micromolar concentrations and have diverse effects on cellular survival, proliferation, and functions[1,2,3,4,5]. The LPLs were discovered first chemically in the mid- to late-1800s, among other organic solvent-extractable components of cells and tissues. Glyceryl-LPLs, exemplified by LPA, are members of the phospholipid family that predominates quantitatively among lipid-structural constituents of cellular membranes. Sphingosyl-LPLs were named for their shared sphingoid backbone and represent a quantitatively diminutive family compared to glyceryl-LPLs. However, as shown by the protean biological activities of S1P, sphingosyl-LPLs are one of the most structurally and functionally complex classes of biological mediators. As for our evolving understanding of some other bioactive lipids, the appreciation of crucial roles of LPLs in cellular biology derives from recent observations of their rapid generation from cell membrane precursors, often transient early increases in concentration in relation to cellular responses, and potent effects on critical activities of cells both as intracellular messengers and extracellular mediators.

The current intense focus on LPLs was initiated in part by the discovery that many of their effects as extracellular mediators are transduced by a novel family of highly specific G protein-coupled receptors (GPCRs). GPCRs for LPLs[2,4,5,6] were identified first as immediate-early expression elements of responses of endothelial cells to differentiating stimuli, and thus were designated tentatively the endothelial differentiation gene-encoded receptors (Edg Rs). A recent nomenclature subcommittee of the International Union of Pharmacological Scientists (IUPHAR) has recommended replacing these designations by terms based on ligand specificity and order of discovery (see Table 1). The consideration of LPLs and their G protein-coupled receptors (GPCRs) in this review will be directed principally to descriptions of their distinctive effects as mediators of cellular differentiation, survival, proliferation, and cytoskeleton-based functions in several mammalian systems. There will also be discussions of new findings that provide compelling support for roles of LPLs in several human diseases. Most prominent among these are autoimmunity, coronary artery disease, and some forms of cancer.

## BIOSYNTHESIS OF LPLs

The series of enzymes responsible for separate production of LPA and S1P, which are to be secreted after cellular stimulation, consists of different phospholipases, sphingolipases, and highly specialized lipid kinases (Fig. 1). A different array of intracellular enzymes participates in *de novo* synthesis of precursors to be stored in cellular membranes as the sources of LPA and S1P released from stimulated cells (Fig. 1). Phospholipid-rich plasma membrane microvesicles are released from activated platelets, leukocytes, epithelial cells, and some tumors. After sphingomyelinase conditions these membrane microvesicles, phospholipase C (PLC)- and/or PLD-dependent mechanisms liberate phosphatidic acid, which is converted to LPA by secretory PLA2 and possibly other phospholipases[7,8]. Analogously, much of the secreted S1P is generated from cell membrane stores of sphingolipids by the sequential actions of sphingomyelinases, ceramidase, and sphingosine kinase (SK) (Fig. 1)[9]. A novel pathway for generation of extracellular S1P has been described very recently. In some circumstances, SK is secreted and converts extracellular sphingosine to S1P[10]. The relative importance of this source of S1P has not yet been evaluated systematically. PLA2 and SK are the dominant rate-controlling enzymes in their respective synthetic pathways[7,9]. However, concurrent degradative activities of a series of lysophospholipases, lysolipid phosphatases, and acyltransferases, and a S1P-specific lyase contribute significantly to the courses of appearance and net maximal concentrations of

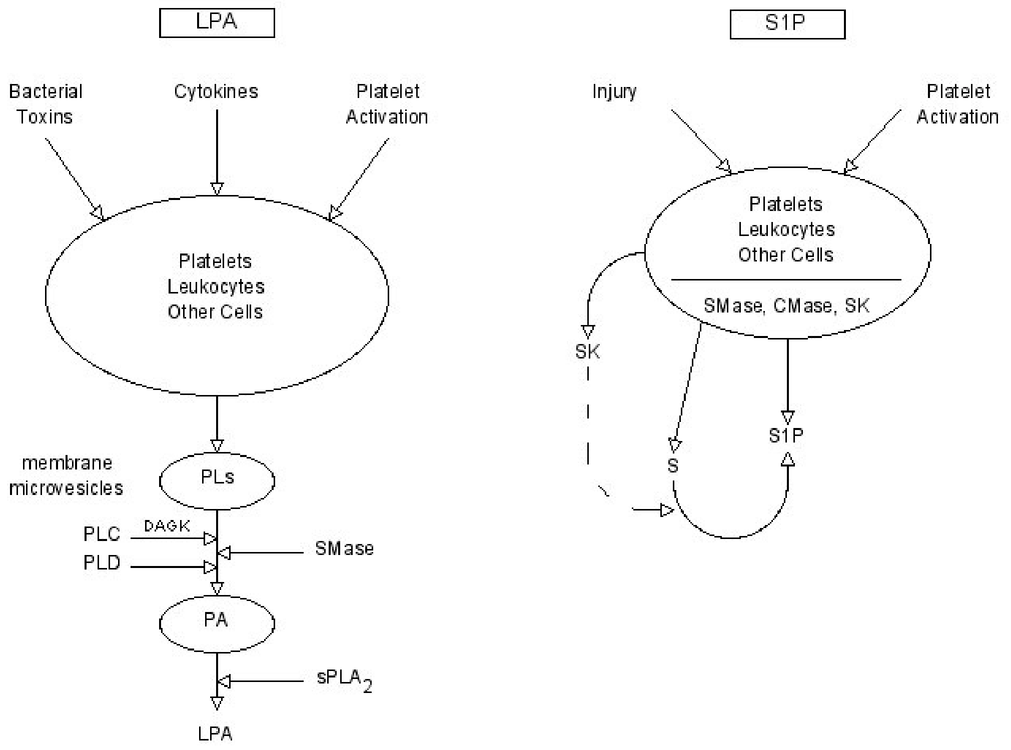

**FIGURE 1.** Cellular generation and release of lysophospholipid mediators. PL = phospholipid, PLC = phospholipase C, PLD = phospholipase D, DAGK = diacylglycerol kinase, sPLA2 = secretory phospholipase A2, PA = phosphatidic acid, SMase = sphingomyelinase, CMase = ceramidase, S = sphingosine, SK = sphingosine kinase.

LPA and S1P attained in any reaction[6]. The dependence of tissue and fluid concentrations of LPA and S1P on multiple LPL-generating and LPL-metabolizing enzymes suggests that many different genetic defects will alter their respective bioavailable levels, with functional significance for numerous organ systems.

## CELLULAR SOURCES, TRANSPORT, AND DELIVERY OF LPLs

LPA was detected by its vasoactivity in incubated normal plasma in 1954, tentatively identified in 1960, and definitively characterized structurally and as a serum vasoactive and platelet-active factor in 1978–1979[11,12]. The serum concentration of LPA is micromolar, in contrast to nanomolar levels in fresh plasma, confirming that platelets are a major source of LPA[13]. Macrophages, some other types of leukocytes, epithelial cells, ovarian cancers, and some other tumors also produce LPA, resulting in high intracellular concentrations and plasma levels up to 50 μM in some inflammatory and neoplastic diseases. The interactions of LPA with various intracellular lipid-binding proteins have not been studied to date. Extracellular LPA is bound by serum albumin with an apparent Kd of 350 nM, which enhances cellular delivery and effective potency[14]. Recent analyses have shown high-affinity binding of LPA to plasma gelsolin, with an apparent Kd of 6 to 7 nM, which delivers LPA to some types of cells more efficiently and with more potent activity than serum albumin[14]. LPA binds to the two L-α-phosphatidylinositol 4,5-bisphosphate ($PIP_2$) sites of gelsolin in competition with $PIP_2$. Cellular delivery of LPA by gelsolin is most effective at concentrations of 1% to up to 10% of those in normal plasma. At concentrations over 10% of those of normal plasma, gelsolin traps LPA and prevents access to cells with an efficiency that may explain the lack of effect of plasma LPA on endothelial cells normally. Thus plasma gelsolin may carry LPA in an inactive state. LPA may then be delivered to cells in an active state when gelsolin levels drop in plasma and tissue fluids as a result of dilution and binding to actin released from injured cells. This possibility is supported by findings of gelsolin concentrations optimal for cellular delivery of LPA in fluids of burned tissues and airway secretions of inflammatory lung diseases. Sphingosyl-LPL mediators, such as S1P, are formed during turnover and degradation of membrane sphingolipids by diverse sphingomyelinases and downstream enzymes in numerous types of cells[1]. Plasma and serum concentrations of S1P are in the micromolar range, with extensive binding normally to serum albumin and possibly other proteins, but not gelsolin.

## CELLULAR RECEPTORS FOR LPLs

Two distinct subfamilies of GPCRs bind LPA and S1P specifically and transduce diverse cellular signals by associating with one or more G proteins (Table 1). Based on amino acid sequence identities, S1P1 (Edg-1), S1P2 (Edg-5), S1P3 (Edg-3), and S1P5 (Edg-8) belong to one structural cluster, and LPA1 (Edg-2), LPA2 (Edg-4), and LPA3 (Edg–7) are members of a second structural cluster[5]. Members of both subfamilies range in size from 351 to 400 amino acids, and are encoded by genes in chromosomes 1, 9, or 19 (Table 1). The amino acid sequence of S1P4 (Edg-6) lies between those of the two major clusters by amino acid sequence identity[17]. LPA/S1P Rs (Edg Rs) share other structural features, which as yet have not been linked clearly to function. The N-linked glycosylation sites of the amino-terminus and multiple potential sites of phosphorylation in intracellular regions, typical of all GPCRs, are preserved in the LPA/S1P receptors (Edg Rs). In contrast, the disulfide bond most often formed between cysteines in the first and second extracellular loops of other GPCRs is observed between the second and third extracellular loops of most LPA/S1P receptors (Edg Rs). In LPA2 (Edg-4 R), an alanine replaces the proline typical of the seventh transmembrane domain sequence NPXXY of other GPCRs. An Src homology-2 (SH2) segment exists in the intracellular face of S1P2 (Edg-5), but has not been shown to express characteristic functions. The mRNAs encoding some of the LPA/S1P Rs (Edg Rs) have the AU-rich sequence AUUUA in their 3'-untranslated region, which is an mRNA-stabilizing structure typical of growth-related immediate-early genes.

**TABLE 1**
**S1P and LPA Subfamily of G Protein-Coupled Receptors: Structure and Signaling Mechanisms**

| Human Receptor Nomenclature | Ligand | Kd (nM) | Chromosomal Location | Protein Size (# of AAs ) | Gα Subunit Coupling | Signaling Mechanisms | | |
|---|---|---|---|---|---|---|---|---|
| | | | | | | CAMP | Ca | ERK |
| $S1P_1$ (Edg 1) | S1P | 8 | 1p21.1-3 | 381 | i/o | ↓ | ↑̃/- | ↑ |
| $S1P_2$ (Edg 5) | S1P | 27 | 19p13.2 | 354 | i/o, q, 12/13 | ↑ | -/↑ | ↑ |
| $S1P_3$ (Edg 3) | S1P | 23 | 9q22.1-2 | 378 | i/o, q, 12/13 | ↑ | ↑ | ↑ |
| $S1P_5$ (Edg 8) | S1P | 2 | 19 | 400 | i/o | ↓ | - | ↓ |
| $S1P_4$ (Edg 6) | S1P | 15 | 19p13.3 | 384 | 12/13 | ↓ | - | ↑ |
| $LPA_1$ (Edg 2) | LPA | N / A | 9q31.3-34.1 | 364 | i/o, 12/13 | ↓ | ↑ | ↑/↓[a] |
| $LPA_2$ (Edg 4) | LPA | 73 | 19p12 | 351 (382)[b] | q, i | -/↓ | ↑ | ↑ |
| $LPA_3$ (Edg 7) | LPA | 206 | 1p22.3-31.1 | 353 | i/o, q | -/↓ | ↑ | ↑ |

*Note:* ↑: Stimulation; ↓: inhibition; -: no change
[a]Although LPA1 receptors have been shown to stimulate ERK in some cells, many reports also suggest that it is a negative regulator of cell growth.
[b]The number of amino acids (AAs) in the wild type is 351 and in the mutant is 382.

**TABLE 2**
**Human Edg Receptor Distribution and Their Pathophysiological Roles**

| Receptor | Major Tissue Distribution | K/O Phenotype | Expression in Disease States |
|---|---|---|---|
| $S1P_1$ (Edg 1) | Endothelial cells, CNS neurons, many other cell types | Loss of vascular integrity; embryonic lethal due to intraplacental hemorrhage | |
| $S1P_2$ (Edg 5) | Brain, heart | Increased neuronal excitability; myoclonic seizures in some neonates | |
| $S1P_3$ (Edg 3) | Heart, many other cell types | No apparent phenotype; loss of S1P-mediated signaling in $Edg3^{-/-}$ embryonic fibroblasts | |
| $S1P_5$ (Edg 8) | Brain, spleen | | |
| $S1P_4$ (Edg 6) | Spleen, leukocytes, thymus, lungs | | |
| $LPA_1$ (Edg 2) | Brain (periventricular neurons and non-neuronal white matter), heart, placenta, GI tract | Craniofacial abnormalities; defective suckling behavior; 50% neonatal survival | *Decreased* in MS lesions; *decreased* in cancers, including ovarian cancer |
| $LPA_2$ (Edg 4) | Leukocytes | | *Increased* in many cancers, including ovarian, prostate, cervical and uterine |
| $LPA_3$ (Edg 7) | Prostate, heart, brain, kidneys, testes | | *Increased* in certain cancers, including ovarian and prostate cancers |

*Note:* CNS = central nervous system, GI = gastrointestinal, MS = multiple sclerosis.

The amphipathic nature of LPA has prevented completion of accurate binding studies until recently, when it was discovered that sodium vanadate would suppress unspecific binding and provide data similar to that from analyses of S1P binding. The Kd values for Edg Rs range from 2 nM to over 200 nM (Table 1). Each Edg R exhibits a unique pattern of coupling to Gα proteins, which explains in large part the observed differences in utilization of signal transduction mechanisms (Table 1). For example, all Edg Rs that couple to Gi/o signal decreases in [cAMP]i, except for S1P2 (Edg-5), where the pattern is more complex. Similarly, all Edg Rs coupled to Gi/o and/or Gq exhibit an increase in $[Ca^{++}]i$, except for S1P5 (Edg-8). Distinctive patterns of tissue distribution of each LPA/S1P receptor (Edg R) have been mapped principally by semiquantitative PCR techniques, Northern blots, *in situ* hybridization, and Western blots with monoclonal antibodies to multiple substituent peptides (Table 2). Although most LPA/S1P receptors are widely represented in multiple tissues, each is differently distributed in the nervous, endocrine, cardiovascular, and immune systems at sites of their principal effects.

Several GPCRs in the orphan pool, which are not meaningfully homologous with the original Edg subfamily, recently have been identified as specific receptors for biologically active phospholipids (PLs). Although S1P1, S1P2, and S1P3 bind sphingosylphosphorylcholine (SPC) with low affinity, the first authentic SPC receptor was found to be the GPCR previously designated GPR68, or the ovarian cancer G protein-coupled receptor 1 (OGR1). The binding of SPC to OGR1 is high affinity, stereospecific for D-erythro-SPC, and stimulatory for a transient increase in $[Ca^{++}]i$[16]. Although the expression of OGR1 is highest in spleen, blood leukocytes, liver, lungs, small intestines, and testes, little is known of its native physiological functions. That there is such high representation of OGR1 in lymphocytes is of great interest from the perspective of the immune connections of two other newly recognized PL GPCRs. The lymphocyte GPCR, termed G2A, is a specific high-affinity receptor for lysophosphatidylcholine (LPC)[17]. G2A signaling through increases in $[Ca^{++}]i$ and ERK MAP kinase activity enhances T cell migration. The exact immune roles of increases in G2A expression by lymphocytes after prolonged mitogenic or stress stimulation are unknown, but late negative regulation of immune responses seems likely. This possibility is reinforced by the findings that mice, which genetically lack G2A, develop late-onset autoimmunity characterized by lymphoid hyperplasia, expanded sets of B cells and T cells, antinuclear autoantibodies, and immune complex nephritis. The glycosphingolipid D-galactosyl-β-1,1'-sphingosine, or psychosine, which has diverse effects on cell migration and functions normally and in globoid cell leukodystrophy, is the specific ligand for the GPCR designated TDAG8[18]. TDAG8 is encoded by the T cell death-associated gene 8, which is up-regulated during T cell apoptosis and also expressed by several subsets of mononuclear phagocytes. TDAG8 and OGR1 show 41% amino acid sequence identity, and have substantial homology with G2A. A fourth PL GPCR, GPR4, has approximately 50% homology with OGR1, binds SPC with high-affinity, and also recognizes LPC with a relatively lower affinity. GPR4 and OGR1 have different tissue distributions. GPR4 also is highly expressed in lung tissues, and found in substantial amounts in liver, kidney, ovary, and lymph nodes[19]. Thus this newest subfamily of GPCRs for PLs is heavily expressed by different immune cells and may transduce important immunoregulatory effects of several complex PLs.

## *IN VITRO* CELLULAR EFFECTS OF LPLs: GENERAL CONSIDERATIONS

The principal biological activities of LPA and S1P are as extracellular mediators, which have two basic types of effects (Table 3)[5]. The first are growth-related and include proliferation, differentiation, enhanced survival, and decreased sensitivity to apoptosis of diverse types of cells. The second are cytoskeleton-based functional effects, which include shape change, altered adherence, chemotaxis, contraction, and secretion. Stimulation of proliferation of many different types of cells by both LPA and S1P, through a Pertussis toxin-inhibitable mechanism, is the defining activity of LPLs. A complex set of LPL-initiated signaling pathways results in increases in intranuclear levels of the Ras-dependent ternary complex factor (TCF) and the Rho-dependent serum response factor (SRF), which together bind to and transcriptionally activate the serum response element (SRE) in promoters of many immediate-early response genes critical to cellular proliferation. It is presumed that Edg R transduction of LPL signals to SRE requires association with both Gi/o to recruit Ras and with G12/13 to engage Rho. Confirming evidence comes from the individual abilities of Pertussis toxin inactivation of Gi and of Clostridium botulinum C3 ADP-ribosyl-transferase inactivation of Rho to inhibit proliferative effects of LPLs, and from their greater suppressive effect when applied in combination. In addition to direct nuclear stimulation of cellular proliferation, LPLs also have indirect effects which include increased secretion of autocrine protein growth factors, heightened expression of receptors for protein growth factors, and enhanced expression of plasma membrane-localized protein growth factors such as the heparin-binding epidermal growth factor (EGF)-like growth factor (HB-EGF), which acts on EGF receptors of neighboring cells by a juxtacrine mechanism. In a few types of cells, where LPA elicits an increase in intracellular concentration of cyclic AMP, there is suppression of cellular proliferation[20]. The mechanisms whereby LPLs improve cellular survival are not fully understood, but include reduction in apoptosis. In the few types of cells for which mechanisms of suppression of apoptosis by LPLs have been elucidated, studies have detected both alterations in intracellular levels of effector proteins of the Bcl family and selective inhibition of activity of specific caspases. In T lymphocytes, the protective effects of LPLs are associated with increases in protective Bcl-2, decreases in apoptosis-promoting Bax, and inhibition of caspases 3, 6, and 7, but not 8[21,22].

Cytoskeleton-based functional responses to LPLs include alterations in cellular morphology during differentiation, as typified by LPA-induced rounding of stellate periventricular neurons, and in postmitotic cells, as for the neurite retraction evoked by LPA and S1P (Table 3)[5]. In such responses, stress fiber formation reflects changes in the state of the microfilament network. LPA and S1P evoke focal adhesion kinase activity, activate cell-surface adhesive proteins, and initiate

**TABLE 3**
**Biological Activities of LPA and S1P**

| | | LPA | S1P |
|---|---|---|---|
| a. Growth-Related Effects | | | |
| | Stimulate Cellular Proliferation | Fibroblasts | Fibroblasts |
| | | Renal tubular cells | Monocytes |
| | | Mesangial cells | |
| | | Smooth muscle cells (vascular) | |
| | | Keratinocytes | |
| | | T cells | T cells |
| | | Cancer cells | Cancer cells |
| | Increase Cellular Survival | Macrophages | |
| | | B lymphocytes | |
| | Suppress Apoptosis | Renal tubular cells | Fibroblasts |
| | | Cardiac myocytes | Endothelial cells |
| | | T cells | T cells |
| | | Monocytes | Monocytes |
| | | | Oocytes |
| b. Cytoskeleton-Based Responses: | | | |
| | Cell Morphology | Neurite retraction | Neurite retraction |
| | | Actin cytoskeletal remodeling | |
| | | Myocyte hypertrophy | Myocyte hypertrophy |
| | Cell-cell/Cell-matrix adhesion | Focal adhesion | Fibronectin matrix assembly |
| | | Platelet aggregation | Platelet aggregation |
| | | Leuk.-Endothelial Interactions | Leuk.-Endothelial Interactions |
| | Chemotaxis/kinesis | Tumor cells (transcellular) | Neutrophils (inhibition) |
| | | Endothelial cells | Endothelial cells |
| | Secretion | Neurotransmitter release | |
| | | Protein growth factors | Protein growth factors |
| | Altered electrical excitability; Ion conductance | Neuroblasts | Ventricular myocytes |
| | | Smooth muscles | |
| | | Cerebrovascular myocytes | |
| | Intracellular signaling | | TNF-alpha, adhesion PDGF, proliferation |

*Note:* Leuk = leukocyte, TNF = tumor necrosis factor, PDGF = platelet-derived growth factor.

assembly of a fibronectin matrix on cells. Integrated expression of these responses mediates LPL-elicited platelet aggregation and endothelial interactions with platelets and leukocytes. These and other related events are crucial to cellular chemotactic, secretory, and contractile responses to LPA and S1P. The chemotactic response of several cell types is affected oppositely by S1P, depending on which S1P receptor is the predominant transducer. S1P1 and S1P3 signal chemotactic responses and amplify those from other growth factors, whereas S1P2 signals inhibition of growth factor-evoked chemotaxis. The principal difference in signaling pathways, to which the opposite responses are attributable, is stimulation of the small GTPase Rac by S1P1 and S1P3, in contrast to inhibition of Rac by S1P2.

The principal current obstacle to better understanding the mechanisms underlying the cellular effects of LPLs is the absence of bioavailable and potent pharmacological agonists and antagonists specific for each LPA/S1P (Edg) R. A wide range of analogues and other variants of LPA and S1P have been synthesized for studies of the structural determinants of activity of the parent compounds[6], but most have the same undesirable physicochemical properties as LPA and S1P, and none is a significantly more potent agonist or full antagonist of mammalian receptors. Limited applications of antireceptor antibodies, biochemical inhibitors of characteristic signaling pathways, and genetic approaches, such as overexpression of one receptor or antisense ablation of one receptor, have been useful in the early phases of research, but have not delineated use of individual receptors by most native cells nor been helpful for *in vivo* investigations.

The capacity of S1P to act as a potent intracellular messenger was suggested initially by its compartmentalized generation and localized high concentration in cells responding to protein growth factors[23]. That inhibitors of the sphingosine kinase (SK), which controls synthesis of S1P, suppressed transduction of signals only from some receptors for protein growth factors selectively, and that exogenous S1P reversed this suppression, supported a role for S1P as an intracellular messenger. Similar, but less convincing, data implicate LPA as an intracellular messenger in other instances of cellular signaling.

## EFFECTS OF LPLs ON SURVIVAL, PROLIFERATION, AND FUNCTIONAL ACTIVATION OF T CELLS

Most of the initial studies designed to elucidate the contributions of LPLs in immunity and inflammation have focused on T cells because of their central role in adaptive immunity, chronic inflammation, allergy, and autoimmunity. In addition, T cells are a valuable model for analyses of the proliferative responses evoked and regulated by LPLs. LPLs enhance T cell proliferation and some other responses by the same four often-interactive mechanisms observed in other types of cells. The first is Ras- and Rho-dependent enhancement of serum response element (SRE) activity in promoters of immediate-early growth-related genes. The second is induction of T cell production and secretion of one or more T cell–active polypeptide growth factors. The third is sensitization of T cells to the effects of T cell–selective growth factors. The fourth mechanism, which has been appreciated increasingly as studies of LPLs are extended to a wider range of cell types, is inhibition of proliferation. This phenomenon was observed first in some myelocytes in which LPA increased intracellular concentration of cyclic AMP ([cAMP]i). The results of recent studies of the roles of LPLs in survival and apoptosis of T cells and some other immune cells have revealed alterations in the concentration, localization, or activity of functionally relevant cellular proteins critical to endogenous control of proliferation.

In the initial studies, LPA and S1P had striking effects on T cell susceptibility to apoptosis due to alterations in cellular levels of proteins of the Bcl-2 superfamily and of the caspase array[22]. LPA and S1P also increased T cell sensitivity to diphtheria toxin (DT) as a result of enhanced T cell expression of the receptor for DT, which is HB-EGF[24]. As for many types of T cell tumor lines, cultured Tsup-1 cells of a human $CD4^{+}8^{+}3^{low}$ lymphoblastoma line resembling “double-positive” thymocytes express Edg-2, -3, -4, and –5 Rs, but not Edg-1 R, as determined by both RT-PCR analyses and Western blots. Tsup-1 cell apoptosis was induced by antibodies to CD2, CD3 plus CD28 in combination, Fas, and by cell-permeant ceramide, and was assessed by morphological characteristics, increases in end labeling of free 3'-OH groups of DNA, and release of radioactively labeled fragments of DNA. At $10^{-10}$ to $10^{-7}$ M, both LPA and S1P protected Tsup-1 cells from apoptosis evoked by antibodies to surface proteins[22]. In contrast, S1P but not LPA suppressed apoptosis elicited by C6-ceramide. The failure of LPA to prevent ceramide-induced apoptosis of Tsup-1 cells was partially due to suppression by ceramide of the expression of Edg-2 and –4 Rs, but not Edg-3 and –5 Rs. At $10^{-9}$ to $10^{-7}$ M, both LPA and S1P suppressed Tsup-1 cell content of the apoptosis-promoting protein Bax, without altering levels of Bcl-2 or Bcl-xL. That LPA and S1P suppression of Bax was mediated by LPA/S1P Rs (Edg Rs) was shown by selective antisense-mediated reduction in expression of LPA Rs and S1P Rs separately to levels which prevented depression of Bax by LPA and S1P, respectively, and concomitantly blocked suppression of apoptosis by each LPL. At higher levels of S1P, but not LPA, prevention of Tsup-1 cell apoptosis correlated best with inhibition of activity of caspases 3, 6, and 7.

Other investigations of the effects of LPA and S1P on T cell survival revealed striking sensitization of Tsup-1 cells to the action of DT. HB-EGF is a plasma membrane protein of T

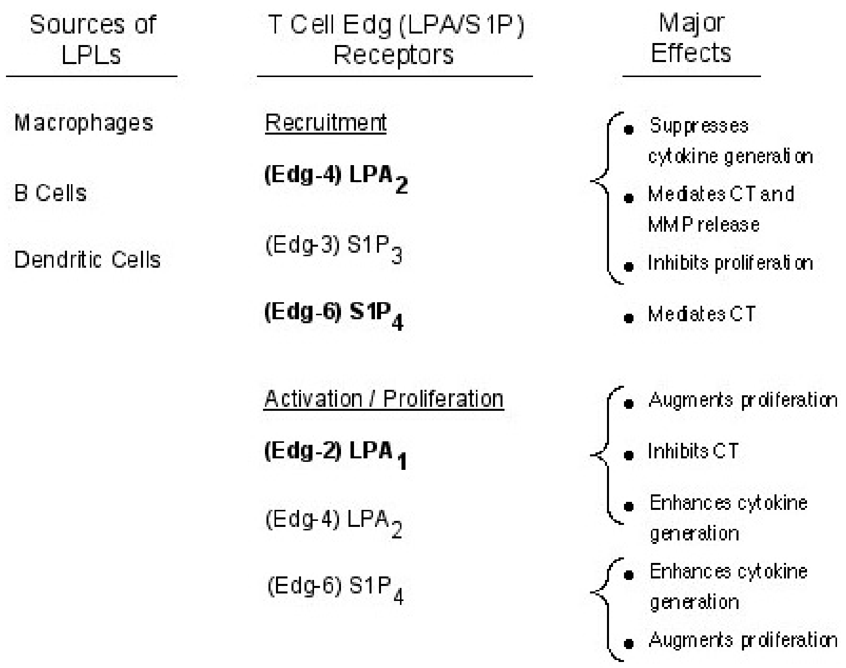

**FIGURE 2.** LPLs in human T cell–dependent immune responses. CT = chemotaxis, MMP = matrix metalloproteinase.

cells, which binds to EGF Rs and matrix proteoglycans, and is the cellular receptor for DT. Under conditions which enhanced sensitivity to DT, LPA and S1P increased Tsup-1 cell expression of HB-EGF[24]. The involvement of increased levels of HB-EGF in LPL enhancement of Tsup-1 cell sensitivity to DT was confirmed by HB-EGF neutralizing antibody blockade of the DT-sensitizing activity of LPA and S1P. Specific inhibitors of pathways of signaling characteristic of Edg Rs reduced LPA and S1P stimulation of both expression of HB-EGF and increased sensitivity to DT. Reductions in expression of LPA and S1P (Edg) Rs by the same antisense strategies used in the earlier studies of LPL protection from apoptosis supported the central roles of these receptors in mediating LPL enhancement of expression of T cell HB-EGF. Thus LPL stimulation of T cell proliferation is augmented by both suppression of apoptosis and enhancement in expression of endogenous protein growth factors.

In contrast to most human T cell tumors and some sets of mouse splenic T cells, which express all of the LPA/S1P (Edg) Rs albeit at substantially differing levels, human blood T cells bear a very restricted representation of LPA and S1P receptors (Fig. 2). Freshly isolated normal human blood $CD4^+$ T cells (helper/inducer or Th cells) showed predominantly Edg-4 Rs (LPA2) and Edg-6 Rs (S1P4), with traces of Edg-3 (S1P3) in some individuals[25]. $CD8^+$ T cells (suppressor/cytotoxic or Ts cells) from the blood of the same normal subjects had no detectable LPA/S1P (Edg) Rs. After activation with a mitogenic lectin or, to a lesser extent, with a combination of anti-CD3 plus anti-CD28 monoclonal antibodies (MoAbs) capable of stimulating T cell antigen receptors, the pattern of expression of LPA/S1P Rs changes rapidly and significantly (Fig. 2)[26]. The level of LPA2 (Edg-4) Rs in Th cells decreases by approximately 50%, that of S1P4 (Edg-6) Rs also decreases, and S1P3 (Edg-3) Rs disappear, as assessed by semi-quantification of mRNA and by Western blots. There is concurrent appearance of LPA1 (Edg-2) Rs in Th cells at a level less than or equal to that of LPA2 (Edg-4) Rs. Barely detectable levels of LPA1 (Edg-2) and S1P2 (Edg-5) Rs develop in Ts cells. Thus T cell expression of Edg-type LPA/S1P Rs is highly flexible, in relation to the source and state of functional activation of the T cells.

Equally impressive modifications in human blood Th cell functional responses to LPLs are observed after exposure to mitogens (Fig. 2). The responses to LPA have been examined in detail, but those evoked by S1P are still under investigation. Prior to mitogen stimulation, LPA elicits Th cell migration through a basement membrane-like Matrigel barrier by enhancing chemokinetic mobility and augmenting secretion of matrix metalloproteinases (MMPs) of the gelatinase subfamily. This response is presumed to be transduced by the Edg-4 (LPA2) R, as agonist-like mouse anti-LPA2 (Edg-4) R IgG MoAbs elicit migration of freshly isolated Th cells similarly to LPA. LPA has an inhibitory effect on IL-2 generation and secretion by freshly isolated Th cells incubated with a combination of adherent anti-CD3 plus anti-CD28 MoAbs, which again is reproduced by agonist-like mouse anti-LPA2 (Edg-4 R) IgG MoAbs. S1P effects resemble those of LPA in the freshly isolated Th cells, but results have not delineated the respective roles of Edg-3 (S1P3) and Edg-6 (S1P4) Rs (Fig. 2). The function-directed activities of LPA are reversed after mitogenic activation of human blood Th cells. An apparent transductional dominance of the newly induced Edg-2 (LPA1) Rs over the remaining Edg-4 (LPA2) Rs results in a lack of direct stimulation of Th cell migration and instead promotes significant LPA inhibition of Th cell chemotactic responses to chemokine stimulation. This new predominance of Edg-2 R signals over those from Edg-4 Rs results in a striking net enhancement of Th cell generation and secretion of IL-2 and of Th cell proliferation (Fig. 2). The present data for S1P effects on mitogen-activated Th cells, which are presumed to be mediated by Edg-6 Rs, are limited to augmentation of proliferation and modest enhancement of IL-2 secretion.

As the activated human blood Th cells express LPA1 and LPA2 Rs codominantly, but no subset of native T cells bears LPA1 R alone, two sets of Jurkat T cells were generated which expressed predominantly LPA1 or LPA2 Rs by transfection of a series of plasmids encoding sense and antisense messages for these Edg Rs followed by hygromycin selection to stabilize lines, as assessed by Western blots[27]. In the LPA2 (Edg-4) R-dominant Jurkat T cells, LPA evoked migration and suppressed IL-2 production, whereas in LPA1 (Edg-2) R-dominant Jurkat T cells, LPA did not evoke migration, suppressed chemokine-elicited migration, and enhanced IL-2 generation. Thus the effects of LPA in LPA2 (Edg-4) R-only and LPA1 (Edg-2) R-only Jurkat T cells exactly mimicked those observed respectively in freshly isolated human blood Th cells and later in mitogen-activated Th cells (Fig. 2). When viewed in terms of the recruitment and activation stages of Th cell involvement in immune responses, the first effects of LPA and probably S1P are to facilitate Th cell movement to the site of an immune response, while suppressing other reactions, such as cytokine secretion, that might be injurious to normal cells in the path of their migration. After their arrival at the site of an immune response, further Th cell migration capable of reducing the population of necessary effector Th cells is suppressed by LPA and effector reactions including cytokine generation are augmented by LPA (Fig. 2). These opposing effects of LPA transduced by Edg-2 and Edg-4 Rs suggest one advantage of multiple Edg Rs specific for the same ligand in precise regulation of complex cellular responses. Similar opposing functions of Edg-2 and Edg-4 Rs also have been observed in some human ovarian cancer cells (OCCs).

## EFFECTS OF LPLs ON HUMAN OVARIAN CANCER CELLS

As high levels of LPA in plasma and ascitic fluid of patients with ovarian cancer correlate with a poor prognosis, it was considered important to investigate the expression and functions of LPA/S1P (Edg) Rs in human OCCs as compared to nonmalignant ovarian surface epithelial cells (OSE). Analyses of mRNA encoding all LPA/S1P Rs showed that LPA2 (Edg-4) and LPA3 (Edg-7) Rs are the predominant Edg Rs in OCCs and ovarian cancer tissues, and are expressed at far higher levels in almost all human ovarian cancer tissue samples than in matching adjacent noninvolved ovarian tissues (Fig. 3)[28]. In contrast, mRNA encoding LPA1 (Edg-2) Rs is more abundant in OSE than OCCs, and in matching adjacent noninvolved ovarian tissues than in human

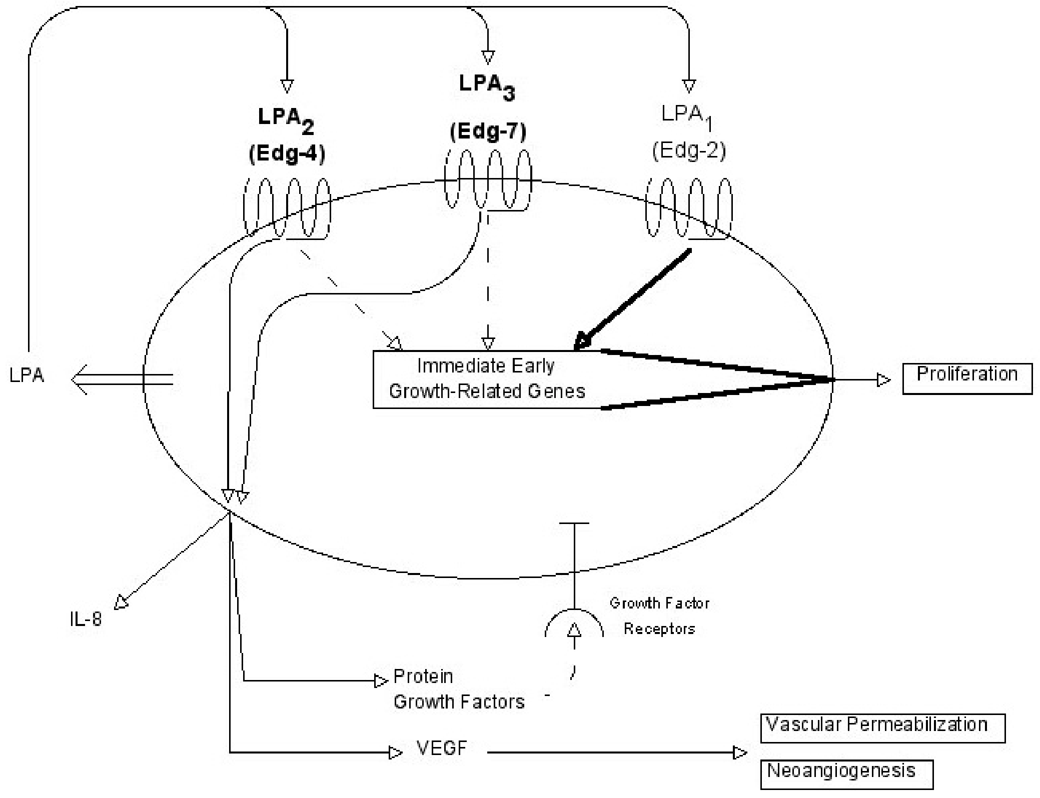

**FIGURE 3.** LPA and LPA receptors in ovarian cancer. Dashed line with arrow = stimulation, heavy solid line with arrow = inhibition, IL-8 = interleukin-8, VEGF = vascular endothelial growth factor.

human ovarian cancer tissue samples. S1P3 (Edg-3) and S1P2 (Edg-5) R mRNA also were consistently higher in OSE cells than in OCCs. Edg-1 R was expressed at similarly low levels in all lines of ovarian cells. Western blots supported the findings of higher levels of LPA2 (Edg-4) and LPA3 Rs in OCCs than nonmalignant ovarian epithelial cells, and higher levels of LPA1, S1P2, and S1P3 Rs in OSE cells than OCCs (Fig. 3). Thus it was expected that OCCs would be more responsive functionally to LPA and normal ovarian cells to S1P.

LPA stimulated proliferation of the OV202 primary line of OCCs, but not IOSE 29 cells, as assessed by increases in uptake of $^3$H-thymidine and cell counts (Fig. 3)[28]. At $10^{-8}$ M, LPA evoked significant mean increases in uptake of $^3$H-thymidine by OV202 cells of 1.7-fold and 2.1-fold, respectively, after 3 and 5 days of stimulation. SRE-luciferase activity of OV202 OCC transfectants, which represents one index of immediate-early gene responses to Edg R signaling, was increased significantly by $10^{-9}$ to $10^{-6}$ M LPA up to a mean maximum of threefold, whereas there was no response of OSE cell transfectants. In contrast, as predicted from the expression profile of Edg Rs, the SRE-luciferase responses to S1P were greater for OSE cells than OV202 cells. OV202 OCC generation of type II insulin-like growth factor (IGF-II), which is a potent mitogen for OCCs, was increased significantly by $10^{-8}$ and $10^{-7}$ M LPA and S1P to maximal levels of approximately tenfold higher than medium alone. LPA also may promote ovarian tumor growth by increasing angiogenesis through stimulation of secretion of vascular endothelial growth factor (VEGF), which is the same protein as vascular permeability factor (VPF). LPA increased secretion of VEGF/VPF by the OVCAR-3 line of human OCCs, up to a mean maximum of fourfold, through a transcriptional activation mechanism, without influencing VEGF/ VPF secretion by OSE cells (Fig. 3)[29]. Pharmacological inhibitors of Edg R transduction suppressed similar LPL stimulation of OCC proliferation, IGF-II generation, and VEGF production and secretion. The capacity of some

OCCs to secrete functionally relevant amounts of LPA suggests that the LPL-Edg R axis may be an autocrine growth and angiogenesis system in ovarian cancer (Fig. 3). The up-regulation of VEGF/VPF also may contribute to the ascites, which is so characteristic of the local peritoneal invasion by ovarian cancer. LPA2 (Edg-4) R and LPA3 (Edg-7) R may be markers for malignant transformation of ovarian epithelial cells, as well as transducers of proliferation by direct nuclear signaling and enhancement of secretion of protein GFs.

## *IN VIVO* BIOLOGICAL ACTIVITIES OF LPLs AND RELATED PLs IN ANIMAL MODELS

LPA, S1P, fluid-phase precursors and homologues of LPA and S1P, and phospholipid ligands of other GPCRs are elevated in at least three different clinical settings: (1) acute lung diseases, such as adult respiratory distress syndrome (ARDS), and acute inflammatory exacerbations of chronic lung diseases, such as asthma, (2) surface epithelial cell injury, as in transcorneal freezing or cutaneous burns, and (3) certain malignancies of which ovarian cancer has been analyzed the most extensively. Of these conditions, animal models have been established or adapted for studies of the roles of LPLs and related phospholipids in lung and ocular tissue trauma and inflammation. An LPA R-dependent mouse model of human ovarian cancer is being developed most recently for investigations of the roles of LPA in the pathobiology of ovarian cancer and of its susceptibility to LPA R antagonist–based therapeutic agents.

Initiation of lung inflammation in guinea pigs by intratracheal administration of lipopolysaccharide induced secretion of LPA-generating type II secretory phospholipase A2 (sPLA2) into bronchoalveolar fluid and accompanying three- to tenfold increases in the concentrations of palmitic acid, total free fatty acids (FFAs), and lyso-phosphatidylcholine (lyso-PC)[30,31]. A specific inhibitor of sPLA2 reduced by a mean of 60% the increases in levels of FFAs and lyso-PC evoked by lipopolysaccharide. Similar increases in the concentrations of FFAs and lyso-PC were attained by administration of guinea pig recombinant sPLA2, in parallel with major decreases in surfactant content of phospholipids. The capacity of lysophospholipase D in lung tissues to convert lyso-PC to LPA is suggested to be one source of increased LPA in pulmonary secretions of injured or inflamed lungs, but was not demonstrated directly. LPA and its active variants cyclic PA and alkenyl-GP were identified at biologically active concentrations in aqueous humor and lacrimal gland fluid from rabbit eyes[32]. The concentrations of LPA and its homologues were increased after corneal injury to levels that stimulated proliferation of keratinocytes isolated from uninjured rabbit corneas.

The phenotypes of mice with a genetic deletion of one LPA/S1P R have revealed LPL functions in some instances (Table 2). The most dramatic abnormalities were seen in S1P1 (Edg-1) R K/O mice, in whom angiogenesis was normal, but vascular integrity was not established due to defective circumvascular migration and adherence of smooth muscle cells and pericytes[33]. The resultant intraplacental hemorrhages led to embryonic lethality in 100% of homozygous S1P1 K/O mice. In contrast, S1P2 K/O mice have a more subtle neural phenotype, characterized by abnormal neuronal excitability and often-fatal seizures. Very modest phenotypic alterations of the facial structures and resultant suckling problems were seen in LPA1 (Edg-2) R K/O mice. Their nervous system appears to be normal functionally, in part due to compensatory increases in neural expression of LPA2 (Edg-4) Rs. No abnormalities were reported for the S1P3 (Edg-3) R K/O mice[34].

## PROTECTION OF CARDIOMYOCYTES FROM HYPOXIA AND OXYGEN FREE RADICALS

S1P and perhaps LPA are vital mediators of cardiovascular organogenesis and functions. S1P1 (Edg-1) Rs have a critical role in development of vascular integrity during embryogenesis. S1P directs endothelial cell migration and intercellular connections, and both S1P and LPA evoke platelet aggregation and elicit proliferation of vascular smooth muscle cells. S1P and LPA alter regional blood flow, including that in the coronary circulation, and modify cardiac inotropy and chronotropy. The S1P and LPA released from mildly oxidized low-density lipoproteins thus may promote atherosclerosis by multiple mechanisms.

Initial profiling of LPL GPCRs in rodent cardiomyocytes and myocardial tissues revealed expression of LPA1 (Edg-2), LPA2 (Edg-4), S1P2 (Edg-5), and S1P3 (Edg-3), but only a low level of S1P1 (Edg-1) and no S1P4 (Edg-6) by Western blots developed with mouse anti-LPL receptor peptide MoAbs[14]. In cultured layers of rat cardiac myocytes, both LPA and S1P evoked serum response element (SRE) transcriptional activity, as assessed by luciferase reporter assays, and hypertrophy, as quantified by increased incorporation of [$^3$H]leucine into proteins. The SRE responses to LPA and S1P were inhibited by both Pertussis toxin and by the C3 exoenzyme inactivator of Rho, and were protein kinase C (PKC) and PI-3 kinase-dependent. S1P was more potent and effective overall than LPA. Gelsolin derived from the cardiomyocytes may serve to regulate the local availability and presentation of LPA. S1P and SPC, which is in plasma at concentrations similar to S1P, also exert a broad range of effects on cardiac myocyte ion channels and electrical activity[6]. S1P and LPA enhance survival of many different types of cells, including endothelial cells, through their respective GPCRs by changing levels of one or more apoptosis-controlling proteins of the Bcl superfamily and by inhibiting activation and/or activities of several caspases.

The capacity of functional LPA/S1P Rs to mediate cardiocytoprotection against injury inflicted by both hypoxia and oxygen free radicals is a new concept and has been examined only recently in several systems. Under normoxic conditions, where neonatal rat cardiac myocytes show a mean viability of nearly 90% after 18–20 h of culture, severe hypoxia reduced viability to 60%, and this was prevented by preincubation with S1P or LPA[35]. In perfused intact mouse hearts, preinfusion of only 10 nM S1P or the S1P-elevating ganglioside GM-1 protected cardiac myocytes from ischemic damage, as shown by improved hemodynamics, decreased release of creatine kinase, and reduced infarct size[36]. S1P and LPA also protect cardiac myocytes and some other cells from injury by oxygen free radicals in several settings.

## PATHOPHYSIOLOGICAL ROLES OF LPLs IN HUMAN DISEASE STATES: OVERVIEW

Elevated concentrations of LPA, lyso-PC, and some other phospholipids have been detected in lesional fluids of several inflammatory and neoplastic diseases. However, only in ovarian carcinoma and possibly other gynecologic cancers have tissue and plasma levels of LPA been increased so consistently as to suggest major pathogenetic roles and possible utility as a biochemical marker of these malignancies[37]. The ability of LPA to stimulate increased expression of tumor cell adhesive proteins and of tumor cell–derived protein growth factors characteristic of the neoplastic state, such as VEGF, transcellular migration, and proliferation, without effects on normal ovarian surface epithelial cells, has contributed to the authenticity of suggestions that plasma levels of LPA represent a useful and functional marker for even early stages of ovarian cancer. Any role of LPA in ovarian and other cancers remains to be proven in further analyses of mechanisms and by large-scale clinical studies of LPA receptor antagonists, involving the effects of different forms of treatment. However, it appears certain that OCCs produce LPA in amounts far exceeding those of any other form of cancer so far examined *in vitro*. Further, OCCs express Edg Rs for LPA qualitatively different from those detected on normal ovarian cells. Although similar quantification of plasma S1P in coronary artery disease has been suggested as an indicator of platelet activation, and thus of severity of activity in chronic ischemic heart disease, no clinical data support this indication.

## ADDITIONAL REFERENCES (SYNOPSIS)

Tokumura, A. (1995) A family of phospholipid autacoids: occurrence, metabolism and bioactions. *Prog. Lipid Res.* **34,** 151–184.

Gaits, F., Fourcade, O., Le Balle, F., Gueguen, G., Gaige, B., Gassama-Diagne, A., Fauvel, J., Salles, J.P., Mauco, G., Simon, M.F., and Chap, H. (1997) Lysophosphatidic acid as a phospholipid mediator: pathways of synthesis. *FEBS Lett.* **410,** 54–58.

## BIOSKETCH

**Edward J. Goetzl** is Robert L. Kroc Professor of Medicine and Professor of Microbiology/Immunology, University of California, San Francisco. Prof. Goetzl conducts research in the areas of immune effector mechanisms, inflammation, G protein-coupled receptors, lipid mediators, neuropeptides, neuroimmunology, and ovarian cancer. Additional research activities include elucidation of roles of lysophospholipid and neuropeptide mediators and their G protein-coupled receptors in T cell development and homing, T cell-dependent immunity and hypersensitivity, cardiac myocyte resistance to apoptosis, and induction of gender-specific cancers.
